# Diagnosis of acute appendicitis in children using urinary 5-hydroxy indol acetic acid and pediatric appendicitis score: A randomized control trial

**DOI:** 10.1016/j.amsu.2021.102274

**Published:** 2021-04-03

**Authors:** Mohammad Gharieb Khirallah, Muhammad Tarek Abdel Ghafar

**Affiliations:** a Departments of Pediatric Surgery, Tanta University, Egypt; b Department of Clinical Pathology, Tanta University, Egypt

**Keywords:** Appendicitis, 5-HIAA, Scoring systems, Diagnosis, Children

## Abstract

**Purpose:**

Acute appendicitis in children represents a common problem. Diagnosis may be difficult due to lack of clinical data. Several scoring systems and laboratory investigations are used for diagnosis. This study aimed to build a model for diagnosis of acute appendicitis in children using urinary 5-hydroxyindoleacetic acid (5-HIAA) and pediatric appendicitis score.

**Methods:**

This study was conducted on 191 children with suspicion of acute appendicitis. They were divided into two groups A and B. Children were evaluated in group A with pediatric appendicitis score, ultrasound, and CRP. In group B children were evaluated in the same manner of group A plus measuring of 5-HIAA.

**Results:**

mean age was 13.3 ± 5.2 years. The mean duration of symptoms was 2.2 ± 1.4 days. The mean level of urinary 5-HIAA was 43.53 ± 24.05 in appendicitis patients in group B. In group A there were 65 cases who had appendectomy. Seventy-five children were operated in group B. Negative appendectomies were found in 13 and 7 cases in groups A and B respectively. Thirteen cases were readmitted in group A with diagnosis of acute appendicitis while seven cases were readmitted in group B.

**Conclusion:**

This combination of urinary 5-HIAA and pediatric appendicitis score builds a model for diagnosis of acute appendicitis in children. This model improves the accuracy of diagnosis of acute appendicitis, reduces both the incidence of negative appendectomies and the incidence of readmission or missed cases in children.

## Highlights

• Diagnosis of acute appendicitis in children is quite difficult.
• Several scoring systems for diagnosis of acute appendicitis in children are present.
• Several urinary biomarkers are used for diagnosis of acute appendicitis in children. None of them is fully accurate.
• Examination of urine sample presents a non-invasive method for diagnosis of acute appendicitis in children.
• 5-HIAA and pediatric appendicitis score highly improves the diagnosis of acute appendicitis in children.

## 1 Introduction

Acute appendicitis represents 16% of all emergency admissions in children and is the most common surgical indication for acute abdominal pain [1]. The global incidence of acute appendicitis is 8–10% and 6–8% in males and females' children, respectively. The most common age is during the second decade of life [2,3].

The diagnosis of acute appendicitis in children especially young may be challenging. The child may not express his/her adequate clinical history or even localize pain. Therefore, some cases may be missed and presented later. Incidence of missed cases ranged from 28% to 57% [4].

Some missed cases may progress to be complicated appendicitis. This dilemma pushed surgeons to operate up on suspected cases. As a result The incidence of negative appendectomy may range from 18to 38% of cases [5].

Several measures have been studied and applied for diagnosis of acute appendicitis in children. Pediatric appendicitis score (PAS) which depends on clinical symptoms and signs together with total leukocytic count and leucocyte left shift is used for diagnosis of acute appendicitis [6,7].

Moreover, imaging studies such as computerized tomography (CT) and abdominal ultrasonography (US) may help diagnosis of acute appendicitis. However, ultrasonography is an operator dependent, and CT exposes the child to ionizing radiation dose. So, these techniques have some limitations [8].

Authors aimed at building new diagnostic model of acute appendicitis in children using spot urinary 5-Hydroxy indole acetic acid (5-HIAA) in combination with PAS.

## 2 Materials and methods

### 2.1 Study design

This is a randomized control trial conducted in a tertiary level hospital during the period from July 2017 to April 2020. It included all children with a suspected diagnosis of acute appendicitis. The study was approved by Institutional Review Board with Approval code 33944/7/20.

**The trial was registered in *clinicaltrials.gov* registry under identifier** NCT04527263. https://clinicaltrials.gov/ct2/show/NCT04527263.

This study has been written in line with Consolidation Standards of Reporting Trials (CONSORT) Guidelines [9].

### 2.2 Eligibility criteria

Two hundred fifty children were presented with the clinical picture of acute appendicitis. Detailed history was taken. This mainly was to exclude cases with history of foods or drugs that may affect level of serotonin in urine such as mono amino oxidase inhibitors. Also, children that were toxic or presented with appendicular abscess or mass were excluded. According to these criteria fifty-nine children were excluded.

### 2.3 Development of randomization sequence

Randomization was developed at the time of initial presentation at pediatric surgery department.

The remaining patients (n = 191) were randomly categorized into two groups:

Group A (n = 95) included patients that were evaluated using our institutional protocol. This protocol consisted of assessment of children using PAS, ultrasound of abdomen and pelvis and serum levels of C-reactive protein.
Group B (n = 96) included children that were evaluated by our institutional protocol. In addition, all children gave a mid-stream urine sample for spot urinary 5-HIAA measurement.

Randomization was achieved using closed envelop method.

### 2.4 Quantitation of urinary 5-HIAA level

From each participant, a random urine sample was obtained to assess the urinary 5-HIAA level using a solid phase competitive enzyme-linked immunosorbent assay (ELISA) technique. The commercial kit was purchased and provided by LDN, DN Labor Diagnosika Nord GmbH & Co. Nordhorn, Germany; Catalog #: BA E−1900. As stated by the manufacturer, the urine samples were methylated to derivatize the 5-HIAA. On the provided microtitre plate, the methylated standards, controls and samples are added. 5-HIAA antiserum was then added and incubated for 1 h. After equilibration, excess free antigen and antigen-antibody complexes are washed out. The affixed bounded antibodies to the solid phase were detected by an anti-rabbit IgG-peroxidase conjugate using TMB as a substrate. The reaction is measured on a microplate reader (Tecan Spectra II, Switzerland), at 450 nm. Quantification of unknown sample concentration is processed by drawing a standard curve with known standard concentrations. The results of 5-HIAA were divided by urinary creatinine and expressed as mg/g creatinine. The inter-assay and intra-assay coefficient of variation were 10.8% and 8.6% respectively.

### 2.5 Clinical outcome

Diagnosis of the acute appendicitis was confirmed by histopathological examination of resected appendix. According to operative findings and pathological reports, the condition ranged from negative pathology of acute appendicitis to different types of acute appendicitis (catarrhal, suppurative, perforated or gangrenous).

Children did not full filling the requirements for diagnosis as acute appendicitis were discharged on symptomatic treatment or after referral to pediatric emergency department.

Readmitted cases were recorded.

### 2.6 Statistical analysis

Statistical analysis was done using IBM SPSS V. 24 (IBM, NY, USA). A descriptive analysis was obtained for patients included in the study. Shapiro-Wilk test was conducted to check for normal distribution of dependent variables, such as urinary 5-HIAA, CRP and histopathological findings. X^2^ test was used to compare the categorical data. Student T-test was used to compare the normally distributed variable between two groups. Mann-Whitney *U* test and Kruskal-Wallis test were used for comparing the non-normally distributed variables of two and more than two groups, respectively. Binary logistic regression was performed to determine the independent predictors for acute appendicitis and to combine 5-HIAA and PAS results in both groups. Pearson correlation was used for correlating the urinary 5-HIAA with other acute appendicitis predictors. Receiver operating characteristic (ROC) curve was constructed to assess the diagnostic efficacy of acute appendicitis predictors. The optimal cutoff value for each predictor was assessed via the Youden index. The value of AUC ranged from 0.5 to 1.0. The ability of a diagnostic test to identify patients with appendicitis was considered optimal as AUC value reached closer to 1.0. P-value <0.05 was considered significant.

## 3 Results

Two hundred-fifty cases were examined during this study. Fifty-nine cases were excluded due to either history of drugs like monoamine oxidase inhibitors administration or appendicular abscess or appendicular mass development.

Accordingly, the study included 191 children with suspected diagnosis of acute appendicitis.

**Fig. 1:**
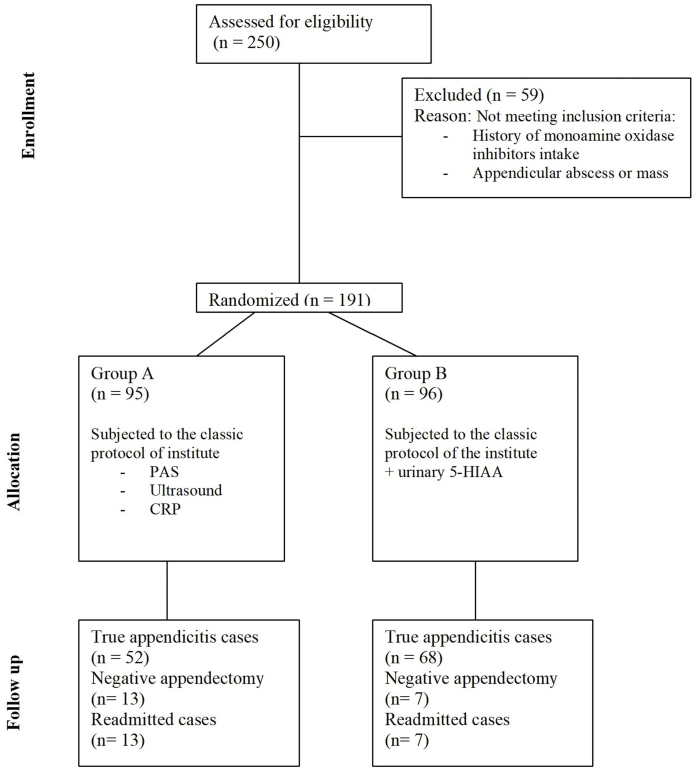
CONSORT diagram showing the flow of participants through each stage of a randomized trial.

Patients’ inclusion and exclusion criteria is presented in Consort Flow Chart (Fig. 1).

### 3.1 Outcomes of both groups

#### 3.1.1 Group A (N = 95)

There were 55 males and 40 females. The median age was 13.3years with range of 4.5–15.3years. Sixty-five children were admitted for appendectomy. The mean duration of symptoms was 2.2 ± 1.4 days Their median of PAS was 6 with range from 5 to 10. The mean serum CRP was 66.3 ± 20.4 mg/l (Table 1)

**Table 1:** Demographic, clinical, laboratory and ultrasound findings in both groups.

	Group A Classic protocol (n = 95)			Group B Classic protocol + 5-HIAA (n = 96)		
	Operated cases (N = 65/95)	Non appendicitis (N = 30/95	P value	Operated cases (N = 75/96)	Non appendicitis (N = 21/96)	P value
Age (mean in years) 13.3 years	12.5 ± 10.4	13.7 ± 9.5	0.634	14.6 ± 9.7	13.6 ± 10.7	0.974
Gender (m/f)%	44/21	11/19	0.324	45/30	14/7	0.342
Duration of symptoms (mean in days) 2.2 ± 1.4	2.2 ± 1.4	2.4 ± 0.6	0.732	2.7 ± 1.1	2.3 ± 0.9	0.562
PAS	6 (range 5–10)	4 (range 0–6)	0.053*	6 (range 4–10)	3 (range 2–5)	0.043*
C-reactive protein (mean mg/l)	66.3 ± 20.4	6.8 ± 3.2	0.045*	45.7 ± 10.6	12.3 ± 7.2	0.057*
Ultrasound - Normal US findings - Signs of acute appendicitis - Non conclusive	- 18/65 - 22/65 - 25/65	- 12/30 - 4/30 - 14/30	0.462	- 22/75 - 40/75 - 13/75	- 11/21 - 3/21 - 7/21	0.629
5- HIAA				43.53 ± 24.05	12.23 ± 9.88	0.001*
Readmitted cases with diagnosis of acute appendicitis	13/30			7/21		0.034*

Histopathological examination of the resected appendix showed that 13 cases had negative pathology of acute appendicitis, 27 cases were catarrhal, 13 cases were suppurative, 7 cases were perforated, and 5 cases were gangrenous (Table 2).

**Table 2:** Histopathological examination of resected appendix in both groups.

	Group A (N = 65/95)	Group B (75/96)	P value
Negative appendectomy	13	7	0.032*
Catarrhal	27	33	0.354
Suppurative	13	19	0.732
Perforated	7	9	0.198
Gangrenous	5	7	0.523

The remaining children (n = 30), their PAS median was 4 with range from 0 to 6 and the mean serum CRP was 6.8 ± 3.2 mg/l (Table 1)

Nine children were diagnosed as gastroenteritis. Three children were diagnosed as having mesenteric lymphadenitis. Eighteen children were discharged on medical treatment with advice to come back to hospital if there was any progression of symptoms.

Thirteen patients were readmitted with clinic picture of acute appendicitis. They were revaluated and were operated (Table 1).

Their histopathological report showed that 9 cases were catarrhal, and 4 cases were suppurative.

#### 3.1.2 Group B (N = 96)

There were 59 males and 37 females. The median age was 12.9 years with range from 4 to 16 years. Seventy-five cases were admitted for appendectomy. The mean duration of symptoms was 2.7 ± 1.1 days. Their median PAS were 6 with range from 4 to 10. The mean CRP was 45.7 ± 10.6 mg/l. Their mean spot urinary 5- HIAA was 43.53 ± 24.05 mg/g creatinine.

Histopathological examination of the resected appendix revealed that 7 cases were with negative appendicular examination, 33 cases were catarrhal, 19 cases were suppurative, 9 cases were perforated, and 7 cases were gangrenous (Table 2).

PAS of the remaining children (n = 21) was 3 with range from 2 to 5. The mean serum CRP was 12.3 ± 7.2 mg/l. The mean spot urinary 5-HIAA was 12.23 ± 9.88 mg/g creatinine (Table 1).

Three cases had gastroenteritis, and 5 cases were diagnosed as mesenteric lymphadenitis.

Seven cases were readmitted with clinical picture of acute appendicitis and underwent appendectomy.

When studying the median levels of spot urinary 5-HIAA in relation to different pathological types of the acute appendicitis, we found that they were 32.6, 47.3, 58.3 and 62.1 mg/g creatinine in catarrhal, suppurative, perforated and gangrenous appendicitis, respectively. Although there were no significant differences in its levels, but the mean level of 5-HIAA was elevated with complicated pathology of acute appendicitis (Table 3).

**Table 3:** Correlation between levels of 5-HIAA and histopathological type of acute appendicitis in group B.

Histopathology (N = 68)	Urinary 5-HIAA	P value
**Catarrhal (N** = **33)**	32.6 (21.3.5–88.2)	0.058
**Suppurative (N** = **19)**	47.3 (18.6–112.0)	
**Perforated(N** = **9)**	58.3 (32.0–90.1)	
**Gangrenous (N** = **7)**	62.1 (33.7–121.3)	

Regarding the inflammatory markers, CRP was significantly higher in the appendicitis group compared to non-appendicitis group (p = 0.047) whereas no significant differences were present in TLC between the two groups. PAS was significantly higher in the appendicitis group than that in the non-appendicitis group (p < 0.001). Moreover, the mean level of urinary 5-HIAA in the appendicitis group was significantly higher than in the non-appendicitis group. There was significant reduction in the incidence of negative appendectomy in group B. There was significant reduction in the incidence of readmission due to appendicitis in both groups.

Multivariable logistic regression analysis of CRP, PAS and urinary 5-HIAA were performed to assess the independent variables for the prediction of acute appendicitis (Table 4).

**Table 4:** ROC curve analysis of variables used for diagnosis of acute appendicitis.

	AUC	P-value	95% C.I	Cut off point	Sensitivity	Specificity	PPV	NPV
**Urinary 5-HIAA**	0.923	<0.001*	0.848–0.968	**> 15 mg/g creatinine**	91.8	87.1	93.3	84.4
**PAS**	0.881	<0.001*	0.797–0.939	**>5**	80.3	74.2	85.9	65.7
**CRP**	0.627	0.005*	0.520–0.726	**>22**	73.8	51.6	75.0	50.0
**5-HIAA** + **PAS**	0.958	< 0.001*	0.881–0.984	**> 3**	93.4%	90.1%	96.3	87.2

### 3.1.3 Development of the model of diagnosis of acute appendicitis in children

**Fig. 2:**
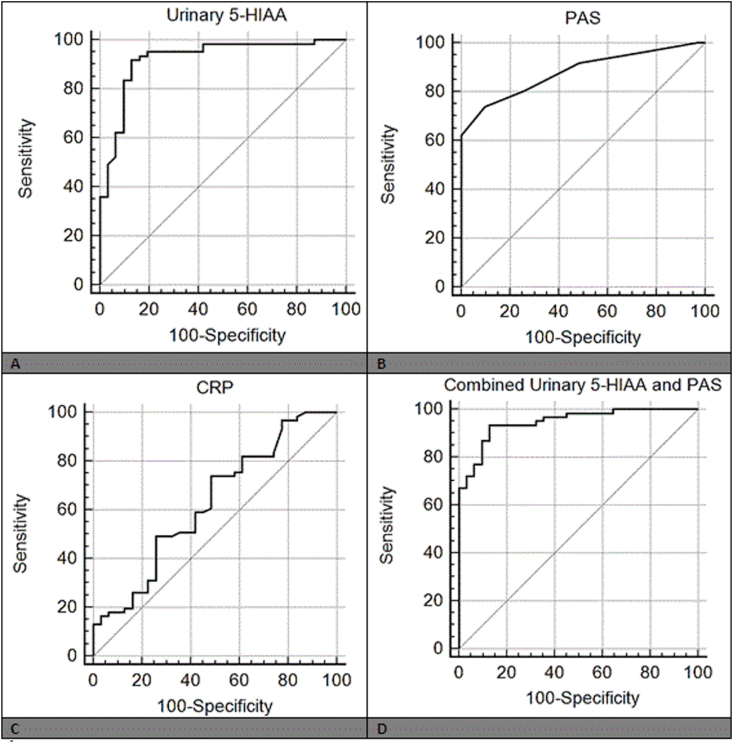
ROC curve analysis of variables used for diagnosis of acute appendicitis.

Accordingly, ROC curve analysis was operated to determine the diagnostic efficacy of CRP, PAS, and urinary 5-HIAA as predictors of the acute appendicitis. The area under curve (AUC) for urinary 5-HIAA was 0.923 (95% C.I: 0.848–0.968). The optimal cutoff value of 5-HIAA is > 16.0 mg/g creatinine with 91.8% sensitivity, 87.1% specificity (Fig. 2A). As regard PAS, the AUC was 0.881 and its cutoff value was >4. At this cutoff, the sensitivity was 80.3% and the specificity was 74.2% (Fig. 2B). CRP presents an AUC of 0.627. At cutoff value > 22 mg/L, the sensitivity was 73.8% and specificity was 51.6% (Fig. 2C). The AUC of urinary 5-HIAA was higher than that of PAS (AUC difference = 0.042, P = 0.357), and CRP (AUC difference = 0.296, P < 0.001).

Pearson correlation revealed that a significant positive correlation existed between urinary 5-HIAA and PAS in the appendicitis group. Therefore, a combined analysis of 5-HIAA and PAS was performed. ROC curve analysis revealed that AUC of combined 5-HIAA and PAS was 0.958 (95%CI: 0.881–0.984) with 93.4% sensitivity and 90.1% specificity (Fig. 2D).

The model of diagnosis of the acute appendicitis in children was developed (Table 5).

**Table 5:** Proposed diagnostic model.

Parameters of model	Score
PAS	
- < 4	- 1
- 4-6	- 2
- > 6	- 3
5-HIAA	
- 0–15 mg/g creatinine	- 1
- < 16 mg/g creatinine	- 2
Interpretation	
- ≤ 2	- Not appendicitis
- 2-4	- May be appendicitis
- 5	- appendicitis

Internal validation of model was performed in the group B cohort. Three parameters were chosen for validation. These parameters are number of cases of cute appendicitis, number of cases of negative appendectomy and number of readmitted cases with acute appendicitis (Table 6).

**Table 6:** Validation of the diagnostic model in cohort group B.

	≤2	2–4	5	pa	Correlation coefficient
True appendicitis	0	7	65	0.035	0.341
Negative appendectomy	0	4	3	0.001	0.194
Readmission	0	7	0	0.004	0.325

a: Spearman rho.

## 4 Discussion

Among many admissions of children with suspected acute appendicitis, negative explorations constituted a considerable incidence. Also, cases that escaped operations and presented later with a complicated clinical picture had a significant figure. This raised attention to develop more clinical scoring systems, perform more laboratory tests or submit suspected cases to more advanced radiological investigations to decrease negative appendectomies. According to the National Surgical Research Collaborative data in UK, the incidence of negative inflammatory process of appendix was about 20.6% [4,10].

Diagnosis of acute appendicitis in children mainly depends on PAS, complete blood count and determination of serum level of C- reactive protein. PAS is a universally applied score system that helps surgeons to put action plan for children with suspicion of diagnosis of acute appendicitis. It is concerned mainly with children who were definitively suffering from acute appendicitis. One of the draw backs of this score was its limited ability in children younger than three years or children that cannot express their clinical picture due to intellectual problems [11].

Clinical picture or laboratory results may simulate other causes of acute abdominal pain and push surgeons to request more advanced radiological methods as ultrasonography, CT or even magnetic resonance imaging (MRI). On the other hand, some of these investigations, had the risk of exposure of children to ionizing radiation doses with increased of 0.2% in risk of the development of malignant lesions. Others need special preparation or sedation, maybe operator dependent or maybe financially expensive and cannot be applicable in many hospitals around world [[12], [13], [14]].

As a noninvasive diagnostic tool, urinary samples provided a method for diagnosis of acute appendicitis. Several biomarkers were measured in these samples. Leucine-rich *α*-2-glycoprotein (LRG), 5-HIAA, IL-6, substance P, calprotectin and bilirubin were used for diagnosis of acute appendicitis. LRG was mainly measured to exclude the diagnosis of acute appendicitis in contrast to the others [15,16].

In the current study authors aimed at building up a diagnostic model of acute appendicitis in children by measuring urinary 5-HIAA in children with suspected acute appendicitis. The main goals were to decrease incidence of negative appendectomy and decrease the incidence of readmission or missed cases with acute appendicitis.

Serotonin is an inflammatory biomarker and one of gastrointestinal neurotransmitter which secreted by enterochromaffin cells [17].

Some studies showed that patients with acute appendicitis had an elevated serum levels of serotonin with sensitivity of about 94% during the first 48 h of symptoms [18].

During current study spot urinary 5-HIAA level significantly increased in acute appendicitis with an excellent diagnostic efficacy (AUC = 0.923) which exceeds the diagnostic efficacy of other acute appendicitis predictors as PAS. Moreover, the diagnostic efficacy of PAS can be improved if combined with spot urinary 5-HIAA.

When the assumed model was activated both the incidence of negative appendectomies and missed cases of acute appendicitis were markedly decreased when compared to classic group. Also, there was decrease in requests of more complex radiological investigations.

In their review Acharya and his colleagues documented that the sensitivity of urinary 5-HIAA was 72% and its specificity was 86% in cases of acute appendicitis. Its area under curve for ROC was 0.88 ± 0.07 [19].

Mentes and his colleague found that urinary 5-HIAA was helpful only for diagnosis of early acute appendicitis and it was significantly elevated during the first 48 h in the inflammatory process and then declined again [20].

Although the results of this study did not show significant differences in the levels of urinary 5-HIAA among different histopathological types of acute appendicitis, there was elevation of its level when correlated to the severity of histopathological results. The urinary 5-HIAA levels were relatively increased in suppurative and perforated appendicitis when compared to the catarrhal type.

On the other hand, Jangjoo found that level of 5-HIAA in urine may not help the diagnosis of early acute appendicitis. However, he found that it was significantly elevated in more complicated cases. Therefore, this study concluded that this test may not distinguish between the normal appendix and catarrhal inflammation [17].

Ilkhanizadeh reported that 5-HIAA in urine was very reliable diagnostic tool of acute appendicitis. Moreover, he found that normal values of 5-HIAA were sufficient to exclude acute appendicitis [21].

Rao and his colleagues found that there was not any significant effect of 5-HIAA measures in patients with acute appendicitis and those with other similar clinical picture. They assumed that the test was invaluable because it could not differentiate between different acute abdominal pain causes [22].

Bolandparavaz and his colleague found that the level of 5-HIAA in urine may decrease or even disappear in complicated cases as gangrene of appendix. They assumed that serotonin secretory cells within the wall of appendix were destroyed in the course of complication [23].

Hernandez had a clinical trial to assess the role of urinary 5-HIAA in cases of acute appendicitis. He thought that the main importance of 5-HIAA was in the cases that had clinical picture of acute appendicitis while other investigations were not conclusive. So, the urinary levels of 5-HIAA can help diagnosis of acute appendicitis [24].

According to available data this study represents the fourth one of series that measures the levels of urinary 5-HIAA among children with clinical picture of acute appendicitis. It is characterized by being randomized control one. The study depended on the combination of measurement of urinary 5-HIAA and PAS to improve diagnostic accuracy of acute appendicitis in children (Table 7).

**Table 7:** Different studies discussing the efficacy of spot urinary 5-HIAA including the current study.

Study	Patient's age (years)	Type of study	Cohort size	Results and conclusion
Current study Khirallah et al.	3–18years	Randomized control study	191 children	- Specificity 87.1, Sensitivity 91.8,PPV: 93.3, NPV:84.4 - Helps the diagnosis of acute appendicitis
Hernandaez et al. [24]	18–70 years	Prospective clinical trial	100 patients	- Specificity: 39–69% Sensitivity: 44–67% PPV: not recorded NPV: not recorded Didn't help diagnosis of acute appendicitis
Jangjoo et al. [17]	18–70 years	Double blinded study	70 adult patients	- Specificity: 81%, Sensitivity: 44%,PPV: not recorded NPV: not recorded - Can't help ruling out acute appendicitis
Bolandparvaz et al. [23]	8–74 years	Case control study	50 healthy control and 60 suspected appendicitis	Sensitivity 84% specificity 88% PPV:92%,NPV: 81%
Mihmanli et al. [25]	16–73 years	Case series study	43 patients	Sensitivity 22%, specificity 93%, PPV: 90%, NPV: 62%
Ilkhanizadeh et al. [21]	Not reported	Case series study	80 suspected cases	Sensitivity 92%, specificity 100%, PPV 100%, NPV: 93% Good test supporting the clinical diagnosis of acute appendicitis
Ozel et al. [26]	9.4 (2–12) years	Case control study	71 suspected appendicitis 34 healthy control.	Sensitivity 70%, specificity 67%, PPV and NPV, were not reported Limited diagnostic tool as a single parameter in children with acute appendicitis.

## 5 Conclusion

The urinary 5-HIAA is a useful diagnostic biomarker for acute appendicitis in children when combined with PAS. It is a noninvasive tool that decreases the need to further advanced investigations. This model for the diagnosis of acute appendicitis in children reduces both the incidence of negative appendectomies and the incidence of readmission or missed cases with diagnosis of acute appendicitis in children.

The limitations and challenges of the current study were a single center study, small sample size, need the availability of marker all the time, and if any lost history of drugs or food may affect the results.

The strengths of the current study were a randomized control trial, and linked to specific age group.

## Sources of funding

There is no sources of funding of that research.

## Ethical approval

Research has obtained ethical approval by Ethical Board Review: Approval code 33944/7/20. Faculty of Medicine, Tanta University.

## Trial registry number

1 Name of the registry: ***clinicaltrials.gov***
2 Unique Identifying number or registration ID: NCT04527263
3 Hyperlink to your specific registration (must be publicly accessible and will be checked): https://clinicaltrials.gov/ct2/show/NCT04527263

## Author contribution

Mohammad Gh. Khirallah: conceptualization, methodology, writing, editing and supervision.

Muhammad Abdelghafar: investigation, formal analysis, reviewing and editing.

## Guarantor

Mohammad Gharieb Khirallah, professor and consultant of pediatric surgery, Tanta University Hospitals, Tanta University.

## Provenance and per reviewer

Not commissioned, externally peer-reviewed.

## Consent for publication

Agree to consent for publication.

## Availability of data and material

The datasets used and/or analyzed during the current study are available from the corresponding author on reasonable request.

## Declaration of competing interest

There is no conflicts of interest.
